# Determining the Area of Ancestral Origin for Individuals From North Eurasia Based on 5,229 SNP Markers

**DOI:** 10.3389/fgene.2022.902309

**Published:** 2022-05-16

**Authors:** Igor Gorin, Oleg Balanovsky, Oleg Kozlov, Sergey Koshel, Elena Kostryukova, Maxat Zhabagin, Anastasiya Agdzhoyan, Vladimir Pylev, Elena Balanovska

**Affiliations:** ^1^ Vavilov Institute of General Genetics, Russian Academy of Sciences, Moscow, Russia; ^2^ Moscow Institute of Physics and Technology, Dolgoprudny, Russia; ^3^ Research Centre for Medical Genetics, Moscow, Russia; ^4^ Biobank of North Eurasia, Moscow, Russia; ^5^ Faculty of Geography, Lomonosov Moscow State University, Moscow, Russia; ^6^ Federal Research and Clinical Center of Physical-Chemical Medicine, Moscow, Russia; ^7^ National Center for Biotechnology, Nur-Sultan, Kazakhstan

**Keywords:** gene geography, ancestry prediction, human population genetics, ancestral origin, machine learning

## Abstract

Currently available genetic tools effectively distinguish between different continental origins. However, North Eurasia, which constitutes one-third of the world’s largest continent, remains severely underrepresented. The dataset used in this study represents 266 populations from 12 North Eurasian countries, including most of the ethnic diversity across Russia’s vast territory. A total of 1,883 samples were genotyped using the Illumina Infinium Omni5Exome-4 v1.3 BeadChip. Three principal components were computed for the entire dataset using three iterations for outlier removal. It allowed the merging of 266 populations into larger groups while maintaining intragroup homogeneity, so 29 ethnic geographic groups were formed that were genetically distinguishable enough to trace individual ancestry. Several feature selection methods, including the random forest algorithm, were tested to estimate the number of genetic markers needed to differentiate between the groups; 5,229 ancestry-informative SNPs were selected. We tested various classifiers supporting multiple classes and output values for each class that could be interpreted as probabilities. The logistic regression was chosen as the best mathematical model for predicting ancestral populations. The machine learning algorithm for inferring an ancestral ethnic geographic group was implemented in the original software “Homeland” fitted with the interface module, the prediction module, and the cartographic module. Examples of geographic maps showing the likelihood of geographic ancestry for individuals from different regions of North Eurasia are provided. Validating methods show that the highest number of ethnic geographic group predictions with almost absolute accuracy and sensitivity was observed for South and Central Siberia, Far East, and Kamchatka. The total accuracy of prediction of one of 29 ethnic geographic groups reached 71%. The proposed method can be employed to predict ancestries from the populations of Russia and its neighbor states. It can be used for the needs of forensic science and genetic genealogy.

## Introduction

Now and then, criminal investigators are faced with the need to infer the ancestral geographical origin of an individual from their genotype. Advances in genome analysis technologies and customization of genotyping arrays have shaped the diversity of currently available platforms for biogeographical ancestry prediction from individual DNA samples. Some of them rely on only dozens or hundreds of SNPs and can predict the continent of a person’s origin (or a large region at best) rather than a specific population (Mehta et al., 2017; Lan et al., 2019; Pakstis et al., 2019). Such platforms are in high demand in countries where individuals of different continental or subcontinental origins constitute the population majority. They are designed to account for human genetic variation at the global rather than local level, even at the cost of sacrificing the number of informative ancestry markers (Phillips et al., 2019). Other arrays can generate more specific predictions, but the markers they use are geographically limited to large regions or subcontinents, like East or South Asia, Oceania, North Africa, Middle East, and Europe (Al-Asfi et al., 2018; Pereira et al., 2019; Lan et al., 2020; Xavier et al., 2020). One of such panels featuring 48 SNPs has proved to be powerful enough to successfully differentiate between three Chinese populations with very different ancestries: Mongol, Uighur, and Han (Jin et al., 2019). However, it is unclear whether the same set of markers can accurately predict the 40 remaining East Asian Chinese populations.

Commercial arrays for genealogy tracing comprise hundreds of thousands of SNPs and produce accurate results, but high costs preclude their use in routine forensic practice, which is limited to dozens or hundreds of SNPs.

Although the arsenal of tools for ethnic geographic ancestry prediction is continuously expanding and more regions are getting covered, one-third of the world’s largest continent remains severely underrepresented. The population of North Eurasia, which spans, among other states, post-Soviet countries, and Mongolia, is incredibly culturally diverse (200 peoples and ten language families) and highly genetically heterogeneous. The immenseness of its genome-wide variation was clearly visible on principal component plots for worldwide population datasets in the early days of SNP-based biogeographic ancestry studies (Li et al., 2008). Using a Humans Origins array featuring 600,000 autosomal markers, Jeong et al. (2019) demonstrated that the composition of the North Eurasian gene pool had been shaped by three major genetic components geographically linked to three ecoregions: forest-tundra, forest-steppe, and steppe. Notably, patterns revealed more than 50 years ago by research studies that relied on classic markers are reproduced today with genome-wide SNP arrays (Balanovskaia and Rychkov, 1990a, Balanovskaia and Rychkov, 1990b; Rychkov and Balanovska, 1992). According to the cited studies, genetic markers characterizing the North Eurasian gene pool occur at different frequencies across populations of North Eurasia. The populations of neighboring regions may not necessarily share them. So, commercial arrays for indigenous ancestry prediction based on dozens of SNPs will provide only rough estimates of European and Asian genetic components for Russian individuals, which is not enough for practical work.

There were attempts to describe the populations of Russia using autosomal STRs and to create an STR-based database for forensic needs. However, the array turned out to have only limited ability to predict ethnic geographic ancestry. The largest dataset representing this region was published in (Stepanov et al., 2011). It consisted of 1,156 samples from 17 populations genotyped for 15 autosomal STRs (Promega PowerPlex16 kit). The dataset represented six Russian cities, nine ethnic groups from Russia, and populations from two other North Eurasian countries (Ukraine and Belarus). The urban populations were shown to be virtually indistinguishable genetically, whereas many ethnic populations differed significantly from each other.

Russia is a vast country with a highly heterogeneous population. At present, there are no SNP arrays to match its diversity. Even the Humans Origins array turned out to be insufficient for the correct differentiation of the population of Northern Eurasia, since it is focused on the world gene pool as a whole. This study was an endeavor to improve the accuracy of biogeographic ancestry predictions for the populations of Inner Eurasia. To that end, the population of this region was divided into 29 ethnic geographic groups that fairly adequately represented its diversity. We determined the range of the most informative autosomal markers that effectively characterize North Eurasian populations and developed a model and software for ancestry inference based on these markers. For the sake of the end user’s convenience, we supplied the software with a cartographic module that shows the most probable area of a person’s ancestral origin on the geographic map.

## Materials and Methods

### Samples

Genotype data was generated from samples representing North Eurasian populations using genome-wide SNP arrays. Most of the analysis was conducted on the data generated by an Infinium Omni5Exome-4 v1.3 BeadChip Kit (Illumina; United States) featuring 4.5 M SNPs. The dataset consisted of 1,883 samples from 266 populations of Russia and its neighbor states. The samples represented 92 ethnic groups from 12 North Eurasian countries: Armenia, Azerbaijan, Georgia, Kazakhstan, Kyrgyzstan, Lithuania, Moldova, Mongolia, Russia, Turkey, Ukraine, and Uzbekistan. The samples were provided by the Biobank of North Eurasia (Balanovska et al., 2016). To avoid terminological confusion when using the words “population”, “people”, “sub-ethnic group”, “geographic group”, “region”, etc., we propose the term “ethnic geographic groups” (EGG) to denote groups of populations that in their totality represent an entire geographic region in such a way that each EGG is relatively genetically homogeneous, but at the same time, its gene pool differs from that of other EGGs.

The study was approved by the Ethics Committee of the Research Centre for Medical Genetics, Moscow, Russia. All procedures performed in studies involving human participants were in accordance with the ethical standards and with the Helsinki declaration (1964).

The written informed consent was obtained from all individual participants included in the study.

### Datasets

Quality control was performed with PLINK 1.9 (Chang et al., 2015). The following filters were applied to create datasets for PCA plots: --geno 0.05 (filters out SNPs with a missing rate over 5%), --maf 0.01 (filters out SNPs with a minor allele frequency below 0.01), --mind 0.1 (excludes individuals with over 10% missing genotype data), and --indep-pairwise 1500 150 0.2 (removes SNPs that are in high linkage disequilibrium with each other). The same filters were applied to create a dataset for SNP selection. The output data were converted to vcf and then to a csv file in which 0 denoted the 0/0 genotype, 1 denoted the 1/0 genotype, and 2 denoted the 1/1 genotype. Finally, missing genotypes were imputed. Imputation is needed because of the inability of a lot of machine learning algorithms to work with missing data. While we have a lot of markers in the initial dataset, we develop the software for the prediction that uses only a limited number of markers. Although haplotype imputation is more accurate, five thousand markers are not enough for this kind of approach. We decided that using a single method for all the data would be more appropriate, so that the training and the test datasets, as well as any newly generated data in the future, would undergo the same preprocessing. Thus, missing genotypes were imputed by replacing a missing value with the most frequent genotype for a given SNP across all 1883 profiles.

After raw data filtering, 51 samples were excluded. Additionally, we removed 19 related samples using KING 2.2.4 software (Manichaikul et al., 2010); all settings were set to default, relatedness was estimated using the --related option. The final dataset consisted of 1813 samples.

### PCA and FST

PCA and FST were conducted using the smartpca tool from the EIGENSOFT software package (Price et al., 2006). Default parameters were used except for the number of iterations for outlier removal in PCA set to 3. The filtered dataset after quality control and pruning described in the previous section was used as input data.

### Machine Learning Algorithms

All machine learning algorithms were used as implemented in the Python 3 Scikit-learn module (Pedregosa et al., 2011). The metrics used are also those implemented in Scikit-learn. All parameters were set to default values if not said otherwise in the Results section. The random seed was fixed for all of the methods to ensure the reproducibility of the study.

## Results

### Workflow Overview

**FIGURE 1:**
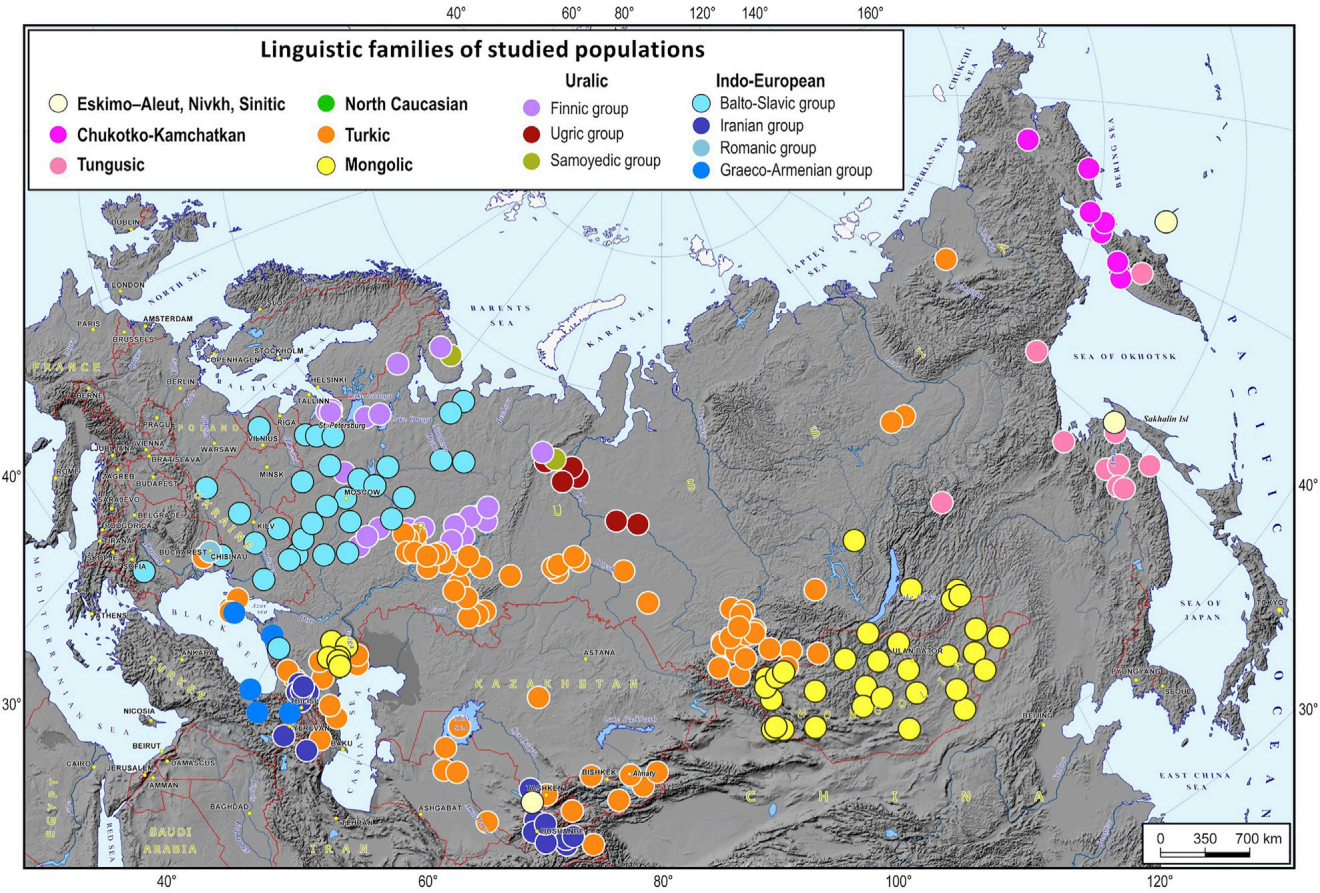
A map of the 266 populations of North Eurasia used for the analysis. Notes. Dots of different colors on the map are languages spoken by the representatives of the studied populations (the color legend is provided at the top of the map).

The dataset included 266 populations from 12 North Eurasian countries: Armenia, Azerbaijan, Georgia, Kazakhstan, Kyrgyzstan, Lithuania, Moldova, Mongolia, Russia, Turkey, Ukraine, and Uzbekistan. The studied populations represent most of the ethnic diversity across this vast territory (Figure 1, Supplementary Table S1). A platform for biogeographic ancestry identification was developed in 5 steps. We started by identifying “ancestry groups”, or “ethnic geographic groups”, i.e., groups of populations that are genetically distinguishable enough to trace individual ancestry. Then, we estimated how many SNPs were needed to differentiate between these groups and chose 5,000 most informative SNPs from the Illumina array of 4.5 M markers. In the third step, we developed a machine learning algorithm for inferring an ancestral EGG. After that, we implemented this algorithm in the original software supplied with a cartographic module for constructing geographic maps of ancestry probabilities. Finally, we validated the proposed method and evaluated its precision.

### Subdividing North Eurasia Into 29 Ethnic Geographic Groups

We aimed to achieve the highest possible geographic resolution of ancestry estimates relying on the limited number of SNPs. There were 266 populations in our dataset (Figure 1), and obviously, it was impossible to genetically distinguish between closely related geographically neighboring populations. This raised the need for clustering the studied populations into groups that would be genetically distinguishable yet relatively internally homogenous. However, the populations were grouped by their genetic characteristics. Below, the groups will be referred to as ethnic geographic because most of them comprised ethnically and linguistically related populations that occupy contiguous territories. In addition to relative genetic homogeneity within a group and apparent differences between the groups, each group had to be represented by at least 25 samples.

**FIGURE 2:**
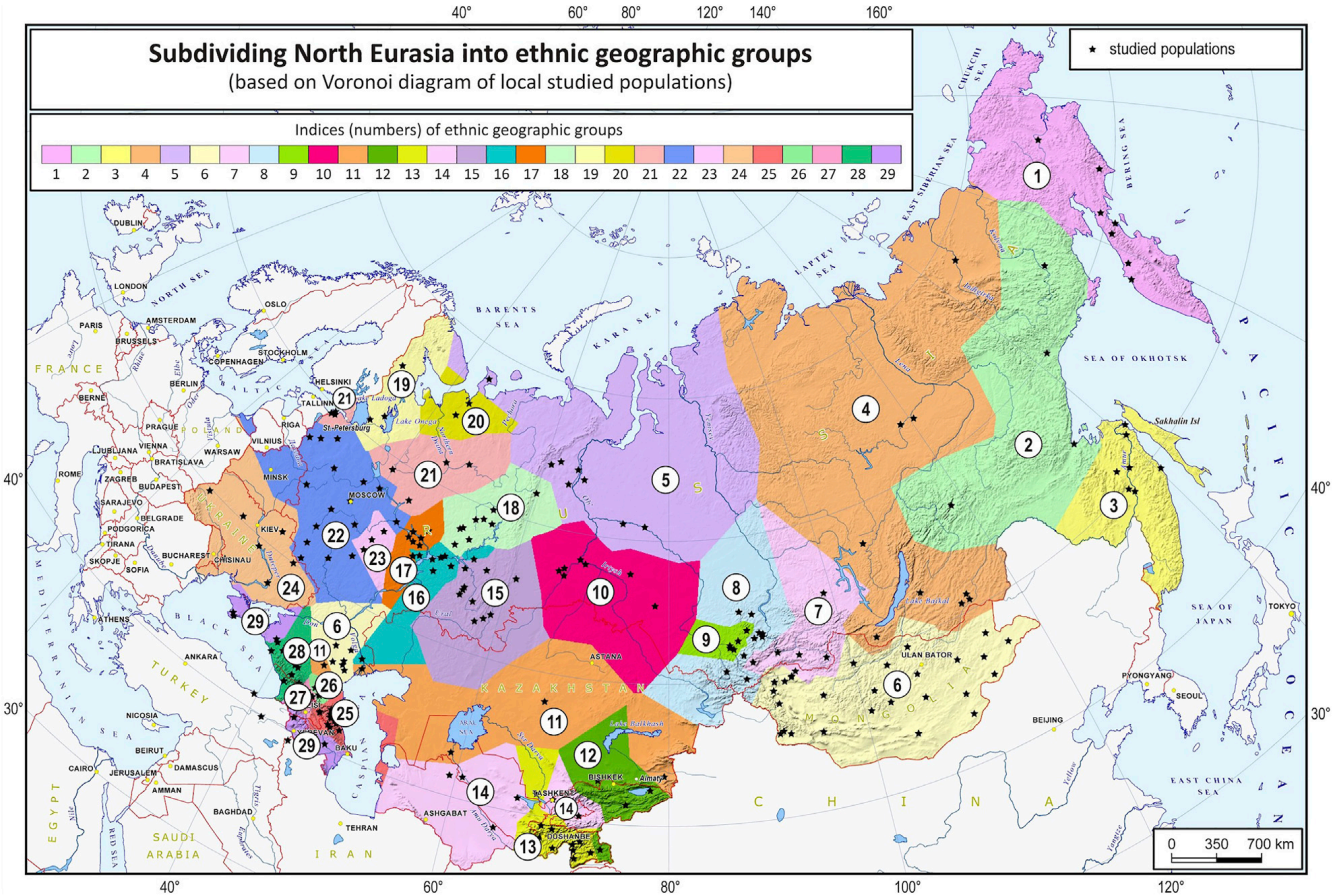
North Eurasia divided into genetically distinguishable ethnic geographic groups. Notes. Colored zones on the map designate areas occupied by the identified ethnic geographic groups. Groups are numbered according to their geographic coordinates. Black stars represent local populations (coincide with the populations in Figure 1).

**FIGURE 3:**
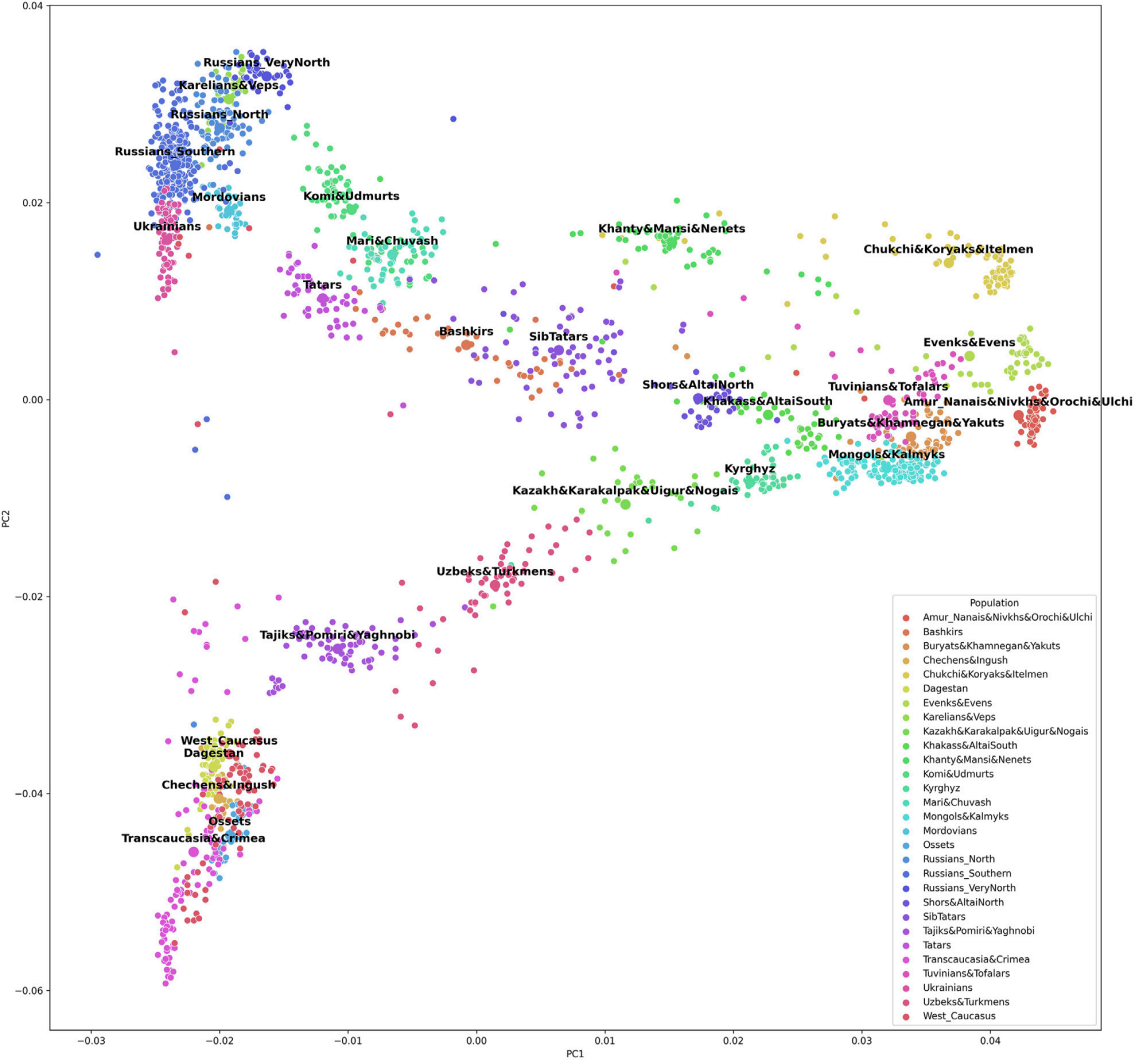
A plot of the first and the second principal components based on the entire 4.5 M SNP panel.

The grouping procedure was previously detailed in (Gorin et al., 2020). Briefly, three principal components were computed for the entire dataset of 1,813 samples (after raw data filtering) using three iterations for outlier removal. For each population, the mean value of each principal component was calculated, and the K-means algorithm was applied to these mean values to partition them into clusters. To obtain clusters with a desired average size, K was set to 30. The method produced 30 imbalanced EGGs (4 samples in the smallest group and 294 samples in the largest). To reduce the imbalance, some of the EGGs were merged while others were broken down into smaller groups so that their size was neither too small (<25 samples) nor too large (>150 samples). The validity of these changes was tested using additional PCA plots for the merged/divided populations and by calculating FST for all pairs of populations. We were not able to merge some of the smaller populations due to their size and genetic difference from other populations, so we removed them from the dataset (40 samples in total). We ended up with 29 groups of populations (EGGs) identified from a set of 1,773 samples (Table 1). Figure 2 shows the area on the map occupied by these groups. Figure 3 and Supplementary Figure S1 show PCA plots for the entire dataset, i.e., 4.5 M SNPs; the color of each sample coincides with the color of the ethnic geographic group it represents.

**TABLE 1:** Populations and sizes of EGGs.

No	EGG	Populations	Size
1	Amur_Nanais&Nivkhs&Orochi&Ulchi	Nanais, Nivkhs, Ulchi, Orochs	55
2	Bashkirs	Bashkirs	44
3	Buryats&Khamnegan&Yakuts	Buryats, Khamnegan, Yakuts	59
4	Chechens&Ingush	Chechens, Ingush	39
5	Chukchi&Koryaks&Itelmen	Koryaks, Itelmens, Kamchadals, Chukchi, Itelmens	75
6	Dagestan	Avars, Kubachins, Dargins, Tabasarans, Laks, Lezgins, Rutuls	74
7	Evenks&Evens	Evens, Evenks	49
8	Karelians&Veps	Karelians, Vepsa	38
9	Kazakh&Karakalpak&Uigur&Nogais	Karakalpaks, Nogais_Astrakhan, Nogais_Stavropol, Uyghurs, Kazakhs	33
10	Khakass&AltaiSouth	Khakass, Altaians	46
11	Khanty&Mansi&Nenets	Khanty, Nenets, Mansi	53
12	Komi&Udmurts	Komi Permyaks, Komi Zyrians, Udmurts, Besermyan	84
13	Kyrghyz	Kyrghyz	43
14	Mari&Chuvash	Chuvashes, Mari	53
15	Mongols&Kalmyks	Mongols, Kalmyks	127
16	Mordovians	Mordovians Moksha, Mordovians Erzya, Mordovians Shoksha	41
17	Ossets	Ossetians	36
18	Russians_North	Russians, Izhora, Vod	81
19	Russians_Southern	Russians, Belorussians	240
20	Russians_VeryNorth	Russians	35
21	Shors&AltaiNorth	Shors, Altaians	37
22	Siberian Tatars	Tatars Siberian	68
23	Tajiks&Pomiri&Yaghnobi	Pomiri, Tajiks, Yaghnobi	72
24	Tatars	Tatars Krayshen, Tatars Kazan, Tatar _Mishar, Tatars from Bashkortostan, Tatars Astrakhan	60
25	Transcaucasia&Crimea	Armenians, Azeri, Tatars_Crimean, Karaites, Turks, Kurds, Ezids, Georgians	113
26	Tuvinians&Tofalars	Tuvinians, Mongols, Tofalars	64
27	Ukrainians	Ukrainians	79
28	Uzbeks&Turkmens	Turkmens, Uzbeks	55
29	West_Caucasus	Adyghe, Kabardinians, Shapsug, Karachays, Abkhazians, Circassians, Abazins, Balkars	87

### SNP Selection

Various methods of SNP selection were tested. The results were compared using an F1-score metric, which is a harmonic mean of precision and recall and therefore ensures a balanced evaluation of predicting power of the model. There were over 817,120 candidate SNPs after raw data filtering, which, considering the small number of samples (1,773), is overwhelming for most feature selection algorithms. At first, we tried the lasso method without univariate feature selection. The resulting F1 score was only 0.42 on average. So, univariate feature selection was performed as a preprocessing step. The chi-square test was applied to each SNP within each class, i.e., EGG. For each class, SNPs with the highest chi-squared values were selected for further analysis. Besides, we experimented with various numbers of SNPs to represent each class and finally settled on 2,000 SNPs. This approach allowed us to reduce the number of candidate SNPs to 50,000–60,000, which is high enough to prevent significant SNPs from being left out and low enough for feature selection algorithms to process the dataset.

For further feature selection, various models were tested. To choose the best feature selection model, we trained a few logistic regression models with identical parameters on the samples of the selected SNPs. The F1 scores obtained by the models were averaged between EGGs and compared to each other. The first tested model was the lasso method without univariate feature selection, which produced an average F1-score of 0.42. By applying the chi-square test, we were able to increase the score to 0.62. Further improvements were achieved by adding size-appropriate weights to classes (EGGs) during model training; this produced an F1 score of 0.65. The procedure was severely affected by overfitting, so we tested the models with less tendency to overfit. The best result was demonstrated by the ExtraTrees classifier less affected by overfitting due to the randomness of the algorithm. Besides, ExtraTrees assigns a score value to each feature and thus can be used to select SNPs with the best score. By adjusting a score threshold, the number of SNPs that get into the final list can be changed. Using the random forest algorithm, we were able to achieve an F1-score of 0.75.

Stratified k-fold cross-validation was applied to further reduce the influence of overfitting. The dataset was split into five subsets (k folds), and the random forest algorithm was trained on these five subsets. An SNP was included in the final list of selected SNPs if its score was above the threshold value in all five models. This allowed us to increase the F1 score to 0.79.

The model performed well for most EGGs, but there were two EGG pairs and one triplet that were often confused by the algorithm: “Northern Russians” and “Southern Russians”; “Mordovians” and “Ukrainians”; “Kazakh&Karakalpak&Uigur&Nogai”, “Kyrghyz” and “Mongols&Kalmyks”. However, all these EGGs were clearly distinguishable on the PCA plots, so we decided to expand the list of optimal SNPs with extra 100 markers with the highest weight that distinguished EGGs in the pairs. To overcome the problem with the triplet, we added 100 SNPs that distinguished two EGG in the triplet from the third and 100 more SNPs that distinguished the two EGGs from each other. This improved the average F1 score to 0.81.

To determine the optimal number of SNPs to be included in the final list of markers, the described workflow was run several times with various numbers of SNPs. Then the logistic regression model was trained on each of the SNP sublists and the performance of the models was compared based on the F1 score. The F1 plot for different numbers of SNPs chosen for ancestry prediction is shown in Supplementary Figure S2.

As seen from Supplementary Figure S2, 4,000 SNPs should be enough to achieve a prediction close to the best possible prediction that can be generated by this model. However, to compensate for imperfect genotyping, we expanded the list to 5,000 SNPs.

After preliminary experiments, we ran the final SNP selection process. First, we selected 2,000 SNPs for each EGG using the chi-square test. Due to the overlap of these SNP sets, our list was narrowed down to 54,522 SNPs. Then, we used ExtraTreesClassifier with balanced class weights and determined the optimal number of estimators to use with cross-validation (CV) and trained one-*vs*-rest logistic regression. The best results were achieved with 320 estimators. After training the model and selecting SNPs with scores above 0.000027 in all CV splits, we ended up with 4,851 SNPs. Then, we added 400 SNPs from the principal components of problematic pairs and triplets to the dataset. The final list comprised 5,229 selected SNPs. The dataset with 1,883 samples and 5,229 SNPs is available in a PLINK format *via* correspondence; characteristics of the samples are provided in Supplementary Table S1. The flowchart of the final SNP selection process is shown in Supplementary Figure S3.

To check whether the selected SNPs adequately reflected the population structure, we constructed PCA plots based on 5,229 SNPs included in the final list (Supplementary Figure S4 and Supplementary Figure S5). Other PCA plots were constructed for the same population sample using the entire set of 4.5 M SNPs from the Illumina panel (Figure 3 and Supplementary Figure S1). The two sets of plots were compared, revealing similar patterns. There was a greater overlap of some population clusters in the second pair of plots, and the distances between some clusters were shorter. However, a decrease in resolution is inevitable with fewer SNPs. By reducing the number of SNPs 1,000-fold, genotyping is made a lot simpler, while the general pattern of genetic similarities between populations remains the same, and the selected set of SNPs allows ancestries to be inferred.

### Building the Prediction Model

After SNP selection was completed, the best mathematical model (classifier) for predicting ancestral populations was chosen and trained. We tested various classifiers supporting multiple classes and output values for each class that could be interpreted as probabilities, including logistic regression, multilayer perceptron (MLP), different variants of Support Vector Classifiers, Naive Bayesian classifiers, and some types of bagging and boosting random forest methods. Their performance was compared based on the average F1-score in all EGGs in 5 CV splits. The best results were demonstrated by MLP and logistic regression (the average F1-score was 0.81). We made an attempt to tune both models. Adjustment of MLP parameters did not improve the score. By tuning the logistic regression model, a slight improvement of the F1 score was achieved, but the score was lower than that obtained with MLP. However, logistic regression is more straightforward and trains much faster than MLP, so we opted for one-*vs*-rest logistic regression with the following parameters: L2 penalty, C equal to 1, and balanced class weight.

This model was trained on 1,773 population samples using previously selected 5,229 SNPs.

### Developing Software for Ancestry Prediction and Mapping the Results

As a part of this study, we developed software for ancestry prediction. The software named Homeland (available *via* correspondence) consists of 3 modules: the interface module, the prediction module, and the cartographic module.

*The interface module* aggregates data from other modules and translates it into a user-friendly format. The module allows the user to submit genotype samples and returns the result of biogeographic ancestry estimation, which can be subsequently printed out as a report or visualized on a savable map.

*The prediction module* estimates a person’s biogeographic ancestry from a submitted genotype. The result is a set of geographic points with different probabilities of ancestral origin.

**FIGURE 4:**
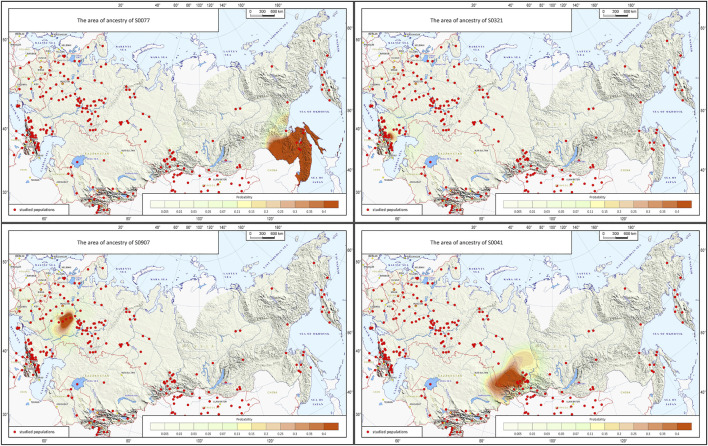
Example of the map generated by the Homeland software.

*The cartographic module* builds JPG maps of probable biogeographic ancestry using the geographic points received from the prediction module and hard-wired settings (cartographic base, scale types, parameters of probability interpolation). The module shows the area of probable ancestral origin predicted from the submitted genotype on the geographic map, which is very convenient for practical work. Besides, the accuracy of prediction can be improved using interpolation: the module will highlight the area where the submitted genotype occurs (Figure 4).

### Validating the Method

To evaluate the prediction power of the model, we trained it on 1,241 population samples from our dataset and then tested it on the remaining 532 samples. The results are presented in Table 2. The EGG prediction heatmap is shown in Supplementary Figure S6. The Figure shows a bright diagonal reflecting the effectiveness of the model: most of the EGG predictions were correct (weighted average precision: 0.85; weighted average recall: 0.84; Table 2). The model made correct predictions about the geographic ancestry of absolutely every individual sample from the following populations: Mari&Chuvash, Ukrainians, Khanty&Mansi&Nenets, Chukchi&Koryaks&Itelmen, Evenks&Evens, Amur_Nanais&Nivkhs&Orochi&Ulchi, Tuvinians&Tofalars, Shors&AltaiNorth (Supplementary Figure S6). There were a few cases when the sample was assigned to 2 EGGs (one correct + one false). These errors occurred with the following groups: Russians_VeryNorth, Karelians&Veps, Russians_North, Russians_Southern, Komi&Udmurts, Mordovians, Buryats&Khamnegan&Yakuts, Mongols&Kalmyks, Khakass&AltaiSouth, Tajiks&Pomiri&Yaghnobi, Ossets, Transcaucasia&Crimea.

**TABLE 2:** Resulting metrics of predictions for each EGG.

	Precision	Recall	f1-Score	Support
Amur_Nanais&Nivkhs&Orochi&Ulchi	1.00	1.00	1.00	12
Bashkirs	0.71	0.77	0.74	13
Buryats&Khamnegan&Yakuts	0.79	0.88	0.83	17
Chechens&Ingush	1.00	0.63	0.77	8
Chukchi&Koryaks&Itelmen	1.00	1.00	1.00	20
Dagestan	0.90	0.90	0.90	20
Evenks&Evens	1.00	1.00	1.00	14
Karelians&Veps	1.00	0.73	0.84	11
Kazakh&Karakalpak&Uigur&Nogais	0.75	0.30	0.43	10
Khakass&AltaiSouth	1.00	0.92	0.96	13
Khanty&Mansi&Nenets	0.94	1.00	0.97	16
Komi&Udmurts	0.96	0.88	0.92	25
Kyrghyz	0.83	0.50	0.63	10
Mari&Chuvash	0.84	1.00	0.91	16
Mongols&Kalmyks	0.82	0.95	0.88	38
Mordovians	0.80	0.67	0.73	12
Ossets	0.86	0.55	0.67	11
Russians_North	0.80	0.35	0.48	23
Russians_Southern	0.75	0.93	0.83	59
Russians_VeryNorth	1.00	0.90	0.95	10
Shors&AltaiNorth	1.00	1.00	1.00	10
Siberian Tatars	1.00	0.65	0.79	20
Tajiks&Pomiri&Yaghnobi	0.81	0.95	0.88	22
Tatars	0.60	0.38	0.46	16
Transcaucasia&Crimea	0.92	0.96	0.94	25
Tuvinians&Tofalars	1.00	1.00	1.00	17
Ukrainians	0.57	1.00	0.73	24
Uzbeks&Turkmens	0.86	0.86	0.86	14
West_Caucasus	0.75	0.81	0.78	26
—	—	—	—	—
accuracy	—	—	0.84	532
macro avg	0.87	0.81	0.82	532
weighted avg	0.85	0.84	0.83	532

False ancestry predictions occurred when the falsely predicted EGG was genetically or regionally close to the actual EGG. For example, 4 individuals from the Russians_Southern population were wrongly recognized by the model as Ukrainians, and Ukrainian ancestry was falsely predicted for 4 Mordovians, 3 Karelians&Veps, 4 Tatars and 3 representatives of the West_Caucasus group (Supplementary Figure S6). Following formal evaluation criteria, this could be interpreted as a reduction in precision. However, all of these falsely predicted EGGs either neighbor the Ukrainian group on the map (Russians_Southern) or inhabit the same region of Eastern Europe so that the false predictions may be due to the high frequency of genotypes inherited from the common ancestor protopopulaton and now spread across this region.

A reduction in sensitivity (low recall) was observed when the sample was assigned to the wrong EGG within the actual ancestral geographic region. Such errors most frequently occurred for the populations of Ural, West Siberia, Central Asia, and Caucasus (Supplementary Figure S6). According to earlier population genetics studies, these territories are highly genetically diverse, which is illustrated by the maps of genetic borders (Pagani et al., 2016; Jeong et al., 2019). This may be due to the vast variety of population sources for these regions. Their contribution differs significantly even between two neighboring populations: being dominant in one population, the contributing genetic component can be very low in another. Therefore, larger sample size and further division of heterogeneous EGGs into more homogenous groups may be needed to ensure more accurate predictions within these regions.

Notably, the highest number of EGG predictions with (almost) absolute accuracy and sensitivity was observed for South and Central Siberia, Far East, and Kamchatka (Supplementary Figure S6).

## Discussion

Forensic science may benefit from a tool for predicting the geographic area of a person’s ancestral origin based on no more than a few thousand SNPs. Studies exploring the gene pools of the western (Western Europe) and eastern (Central and East Asia) poles of Eurasia have generated a massive body of evidence, which, unfortunately, only partly explains the characteristics of the North Eurasian gene pool. They could be better understood using data on the populations of North Eurasian countries that share a history of strong migration flows in the past and present. We determined the range of the most informative autosomal markers in this study that effectively characterize North Eurasian populations and developed a model and software for ancestry inference based on these markers.

Preliminary tests of the proposed model for ancestry prediction allowed us to quantitively evaluate its performance. The analysis of tables generated by the software revealed that the proportion of correct predictions (matches between the actual EGG and the most probable EGG) was 71%. On the maps, the proportion of correct predictions (the actual geographic location being within the most probable predicted region) reached 61% for more likely areas of origin and 81% for less likely areas of origin. Considering the plethora of ethnic geographic groups and the complex population structure of North Eurasia, the proposed method for biogeographic ancestry prediction has demonstrated very good performance.

Merging ethnic geographic groups into larger clusters or expanding the geographic area of probable ancestry improves the accuracy of the model (the proportion of correct predictions) but adversely affects the informative value of the method (geographic precision). This raises the need for further refinement that can be achieved by finding the right balance between accuracy and informative value. Almost absolute accuracy was demonstrated for the majority of EGGs from Siberia, Far East, and Kamchatka. Quite accurate ancestry predictions were achieved for the populations of East Europe, Ural, West Siberia, Caucasus, and Central Asia, and the observed minor deviations in accuracy suggest high genetic heterogeneity in these regions. In our opinion, improvements in prediction accuracy can be achieved by increasing the sample size of the training dataset.

In its current state, the proposed method can be employed to predict ancestries from the populations of Russia and its neighbor states. It can be used for the needs of forensic science and genetic genealogy.

Our method has two limitations: the genotyping approach is expensive, and the method itself has not been optimized for admixed individuals.

We do not propose a genotyping platform that could be used to genotype a DNA sample for the set of 5,000 SNPs. We assume that the sample that the end user has at their disposal has already been genotyped. We propose a method and software to estimate ancestries from the genotype. At the moment, the genotype can be obtained either by using the Illumina Infinium Omni5Exome-4 v1.3 BeadChip or through whole-genome sequencing. We collaborate with another research team that is currently developing a genotyping system for these and some other forensic SNPs. This system will be discussed in a separate publication. Another possible genotyping option is targeted sequencing.

Our method for biogeographic ancestry inference was developed and validated using the set of non-admixed individuals, so the algorithm tends to generate low probabilities of origin from every included ethnic geographic group for genotypes originated from admixed individuals. There are other methods suitable for admixed genetic profiles (Kozlov et al., 2015). Our primary goal was to achieve the highest possible geographic precision of ancestry prediction, and we intentionally focused on non-admixed individuals.

## Data Availability

We followed the regulations of the Russian Federal Law on Personal Data (No. 152-FZ). The generated raw data can be shared *via* personal communication with the corresponding author with the following conditions, (i) the data can be only used for studying population history, (ii) the data will not be used for commercial purposes, (iii) the data will not be used to identify the sample donors, (iv) the data will not be used for studying natural/cultural selections, medical or other related studies. Any additional information required to reanalyze the data reported in this paper is available from the lead contact upon request.

## Supplementary Material

Supplementary Figure S1 A plot of the first and the third principal components based on the entire 4.5M SNP panel.

Supplementary Figure S2 Preliminary analysis of predicting power of the models based on different numbers of SNPs.

Supplementary Figure S3 Flowchart of SNP selection process.

Supplementary Figure S4 A plot of the first and the second principal components based on the selected set of 5,229 SNPs.

Supplementary Figure S5 A plot of the first and the third principal components based on the selected set of 5,229 SNPs.

Supplementary Figure S6 The heatmap of prediction quality: The real EGG *vs*. predicted EGG for the testing dataset.

Supplementary Table S1 Studied samples.
