# Supplementary figures and images for: Determining the Area of Ancestral Origin for Individuals From North Eurasia Based on 5,229 SNP Markers
Source: Front Genet. 2022 May 16;13:902309. doi: 10.3389/fgene.2022.902309 (PMC9149316; doi:10.3389/fgene.2022.902309)

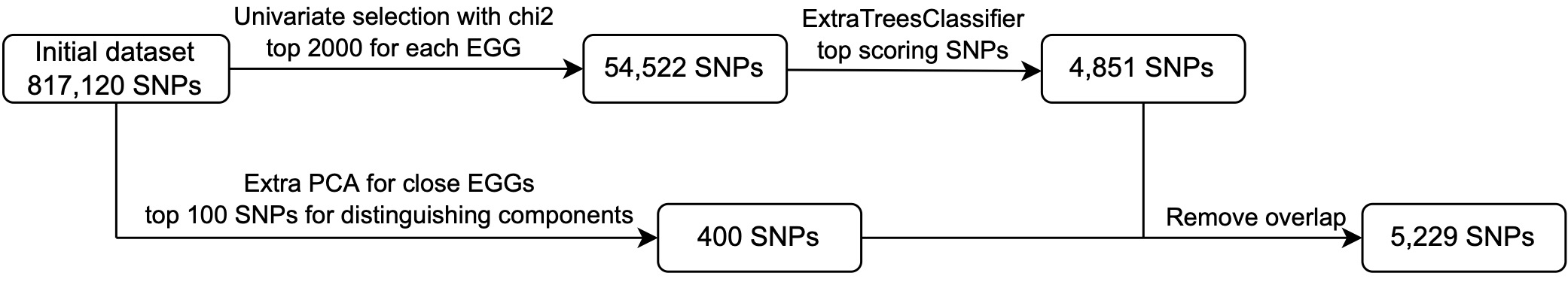

Supplement: Supplementary file 1 [file Image3.JPEG]

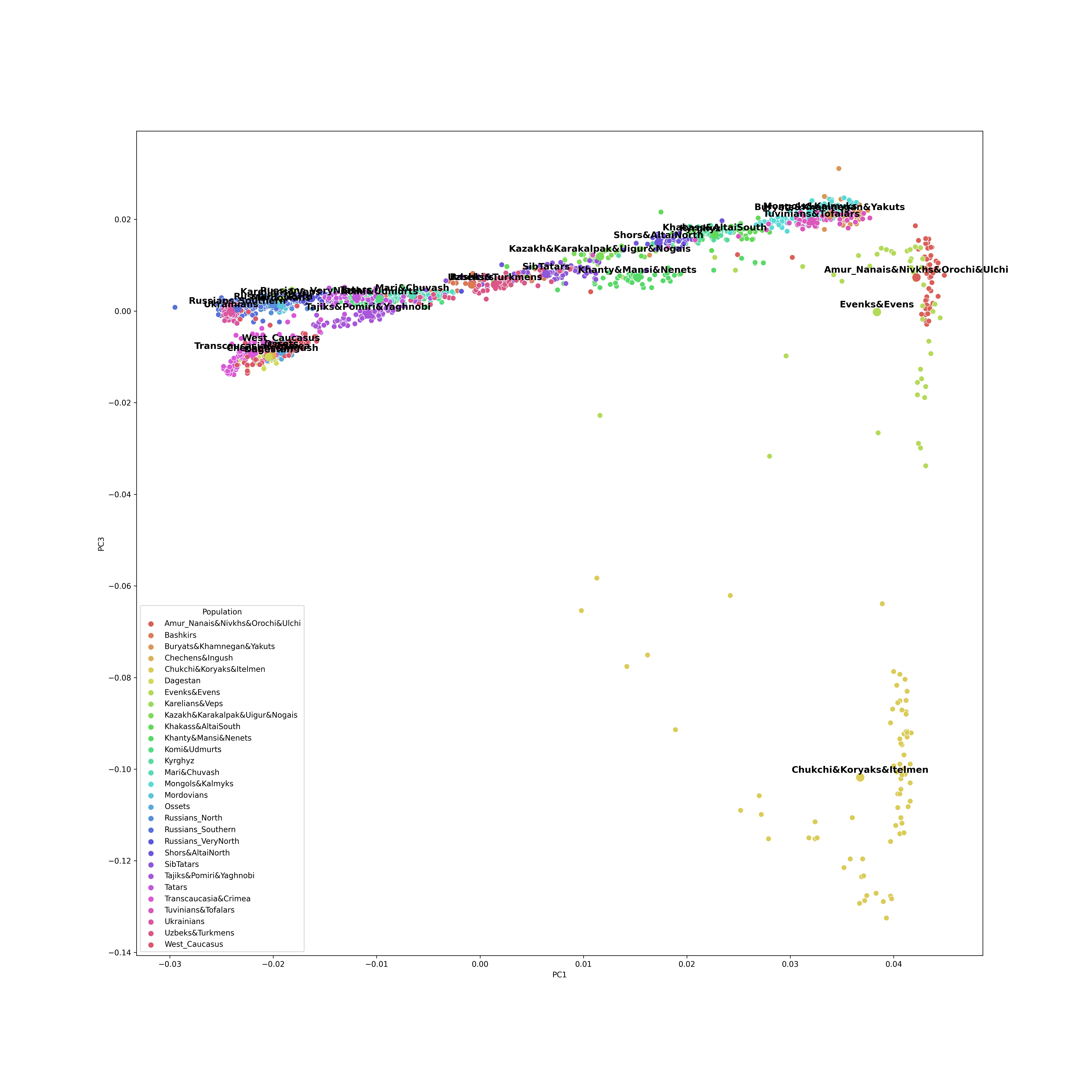

Supplement: Supplementary file 2 [file Image1.JPEG]

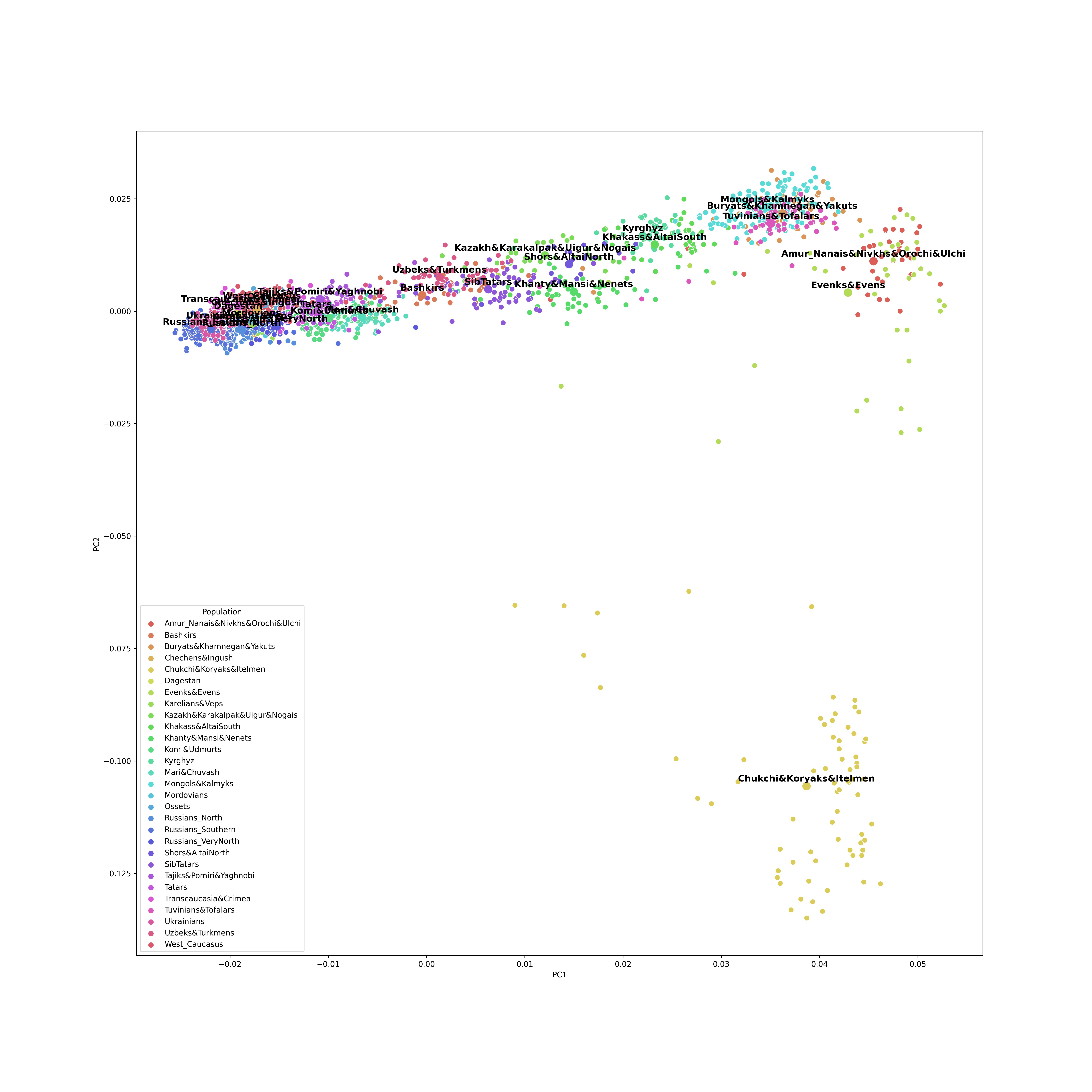

Supplement: Supplementary file 3 [file Image4.JPEG]

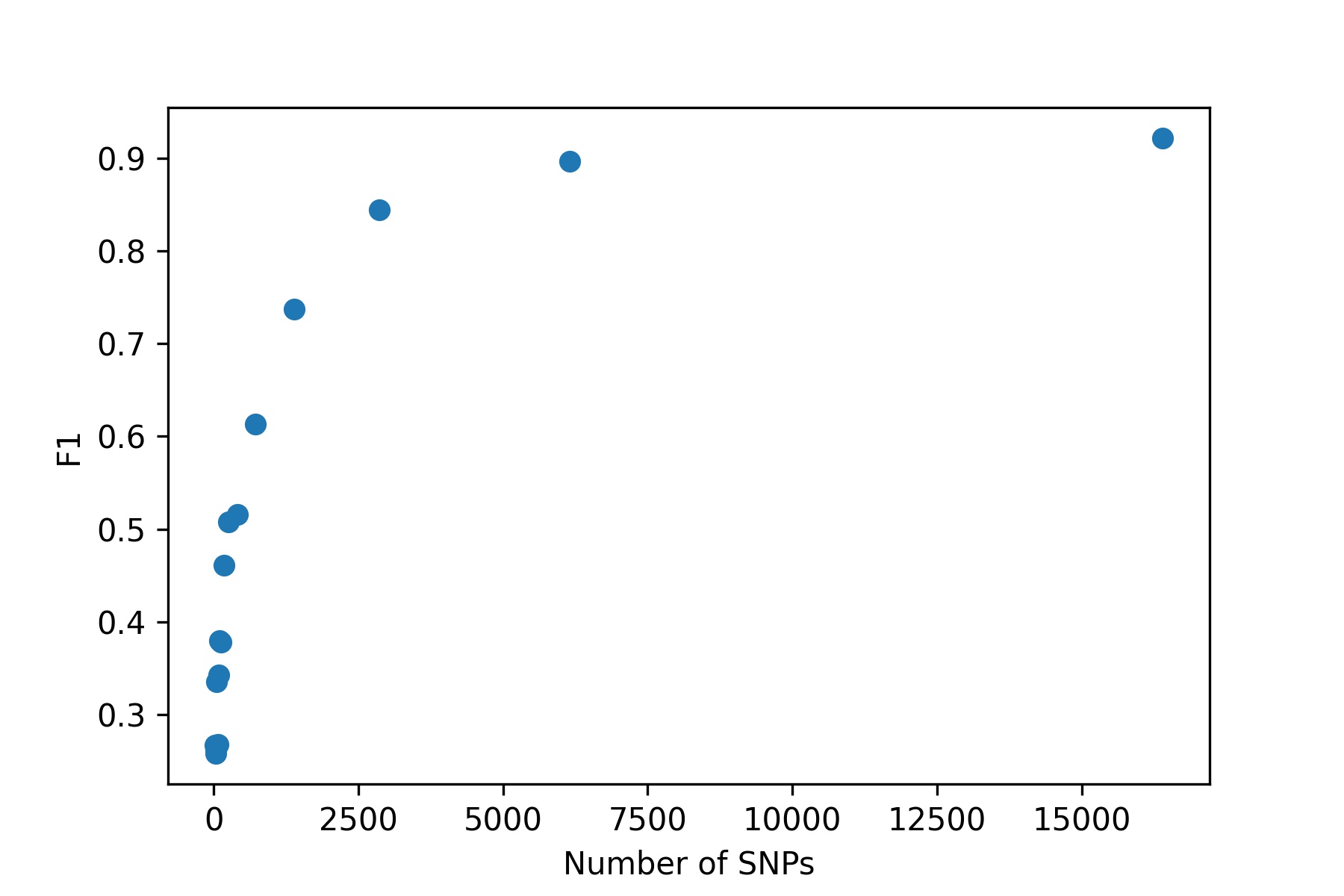

Supplement: Supplementary file 4 [file Image2.JPEG]

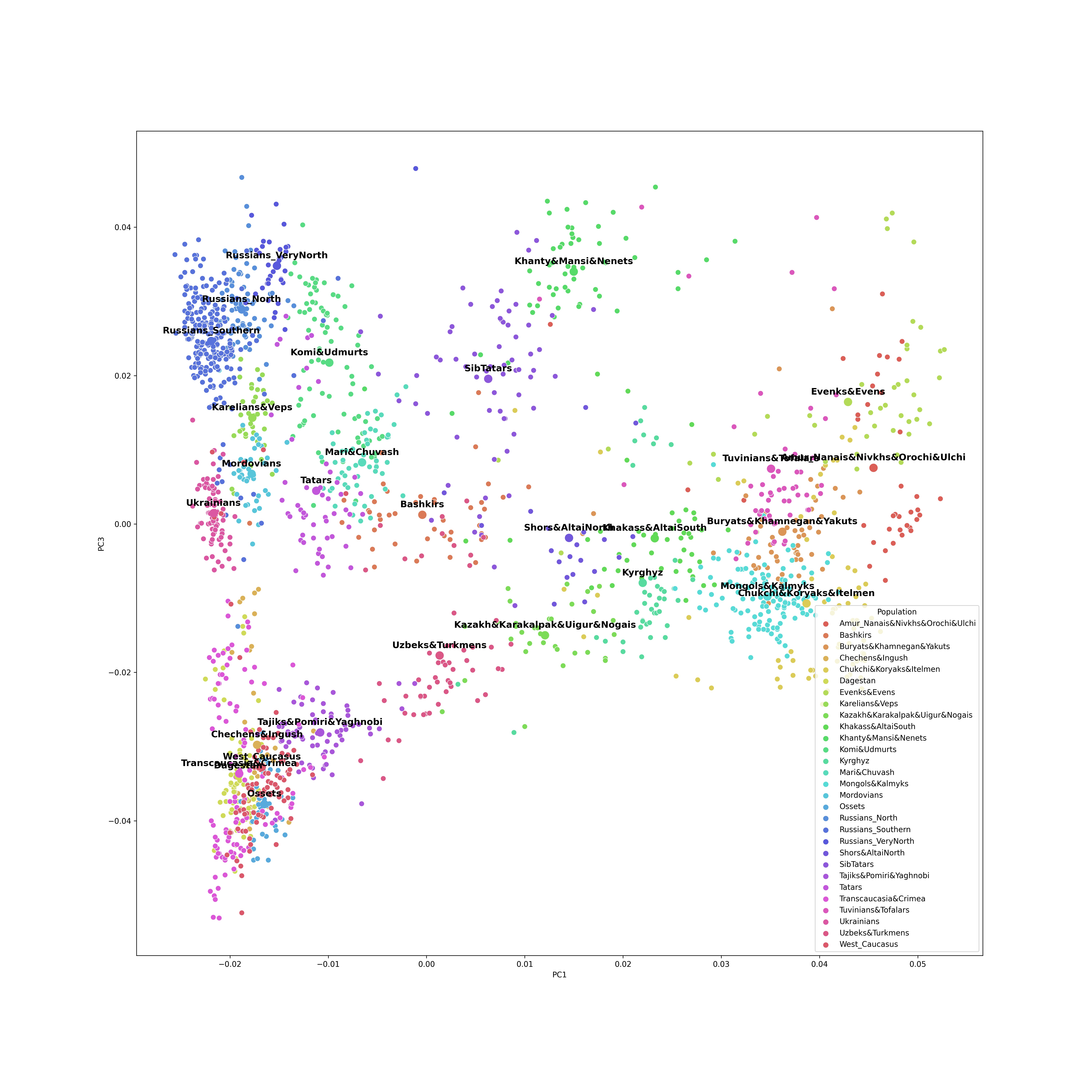

Supplement: Supplementary file 5 [file Image5.JPEG]

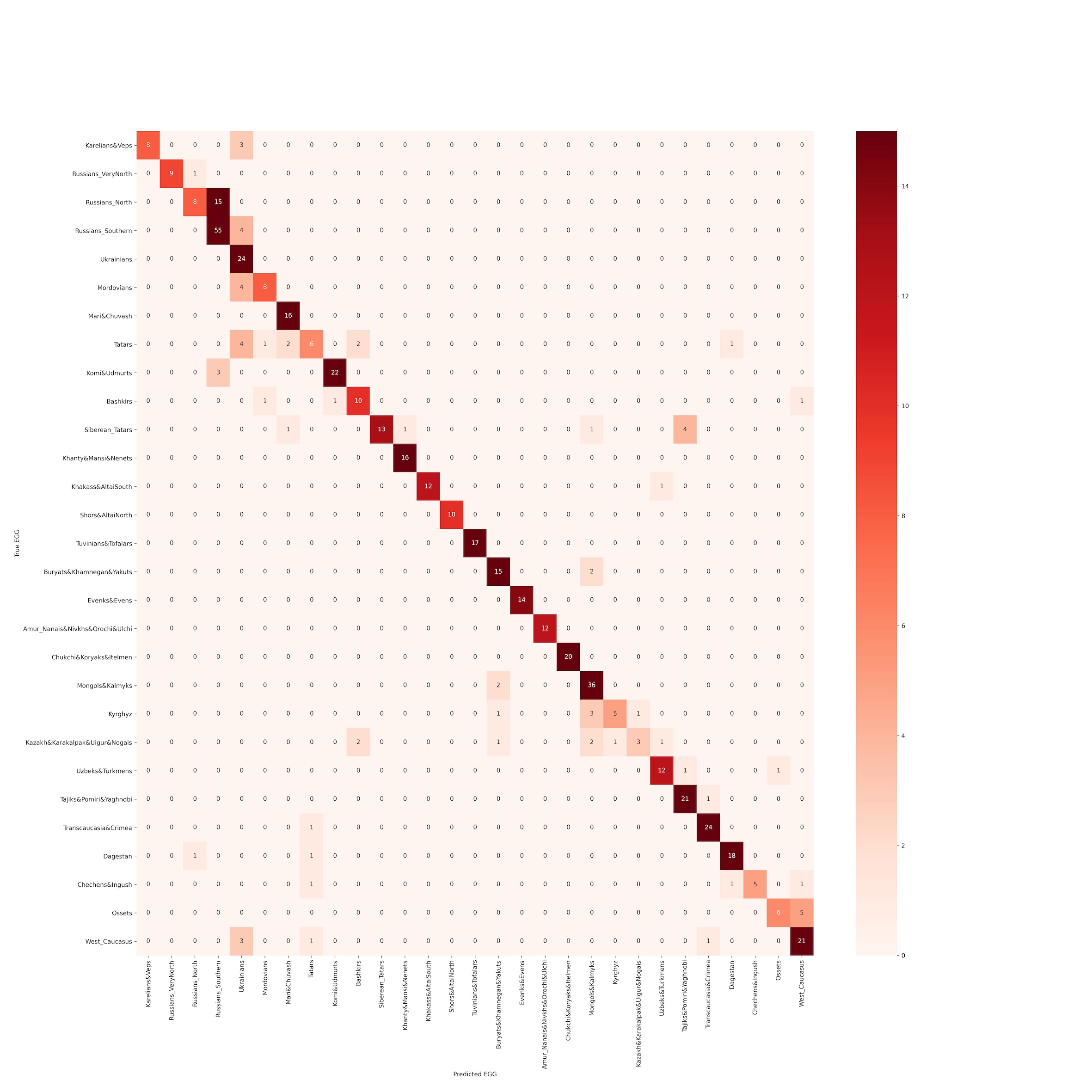

Supplement: Supplementary file 7 [file Image6.JPEG]
